# Using GIS technology to identify areas of tuberculosis transmission and incidence

**DOI:** 10.1186/1476-072X-3-23

**Published:** 2004-10-13

**Authors:** Patrick K Moonan, Manuel Bayona, Teresa N Quitugua, Joseph Oppong, Denise Dunbar, Kenneth C Jost, Gerry Burgess, Karan P Singh, Stephen E Weis

**Affiliations:** 1 Department of Medicine, 3500 Camp Bowie Blvd. University of North Texas Health Science Center at Fort Worth, Fort Worth, Texas 76107, USA; 2 School of Public Health, 3500 Camp Bowie Blvd. University of North Texas Health Science Center at Fort Worth, Fort Worth, Texas 76107, USA; 3 Department of Microbiology and Immunology, 15355 Lambda Drive. University of Texas Health Science Center at San Antonio South Texas Center for Biology in Medicine Bldg, Room 2.100.04, San Antonio, TX 78245, USA; 4 Department of Geography, 1704 W. Mulberry. University of North Texas, P.O. Box 305279 Denton, Texas 76203, USA; 5 Bureau of Laboratories, Texas Department of Health Austin, Texas 78756, USA; 6 Tarrant County Public Health Department, 1101 S. Main St. Fort Worth, Texas 76104, Suite 1600, USA

## Abstract

**Background:**

Currently in the U.S. it is recommended that tuberculosis screening and treatment programs be targeted at high-risk populations. While a strategy of targeted testing and treatment of persons most likely to develop tuberculosis is attractive, it is uncertain how best to accomplish this goal. In this study we seek to identify geographical areas where on-going tuberculosis transmission is occurring by linking Geographic Information Systems (GIS) technology with molecular surveillance.

**Methods:**

This cross-sectional analysis was performed on data collected on persons newly diagnosed with culture positive tuberculosis at the Tarrant County Health Department (TCHD) between January 1, 1993 and December 31, 2000. Clinical isolates were molecularly characterized using IS6110-based RFLP analysis and spoligotyping methods to identify patients infected with the same strain. Residential addresses at the time of diagnosis of tuberculosis were geocoded and mapped according to strain characterization. Generalized estimating equations (GEE) analysis models were used to identify risk factors involved in clustering.

**Results:**

Evaluation of the spatial distribution of cases within zip-code boundaries identified distinct areas of geographical distribution of same strain disease. We identified these geographical areas as having increased likelihood of on-going transmission. Based on this evidence we plan to perform geographically based screening and treatment programs.

**Conclusion:**

Using GIS analysis combined with molecular epidemiological surveillance may be an effective method for identifying instances of local transmission. These methods can be used to enhance targeted screening and control efforts, with the goal of interruption of disease transmission and ultimately incidence reduction.

## Background

The application of molecular analysis to identify specific *Mycobacterium tuberculosis *strains (TB), in combination with traditional surveillance, has yielded insights into tuberculosis transmission [1]. These insights together with a downward trend in tuberculosis in the United States have resulted in the Center for Disease Control and Prevention re-evaluating the TB elimination strategy, and recommending that testing be targeted at specific high risk populations [2,3]. The Institute of Medicine (IOM) also recommended the development of more effective methods for identifying persons with recently acquired infections as an important component of new strategies to limit the spread of tuberculosis [4]. While a strategy of targeted testing and treatment of persons most likely to develop tuberculosis is attractive, it is uncertain how best to accomplish this goal.

Persons with molecularly clustered tuberculosis isolates are assumed to be in the same chain of recent tuberculosis transmission [5,6]. Limited studies have been conducted to evaluate whether these clusters occur in predefined geographical areas [7-11]. If so, then geographically based screening and treatment could be an effective method for TB control programs to identify high risk populations. In this study we seek to determine if we can identify geographical areas with on-going tuberculosis transmission by linking Geographic Information Systems (GIS) technology with ongoing molecular surveillance.

## Methods

This cross-sectional analysis was performed on data collected on all persons newly diagnosed with culture positive tuberculosis at the Tarrant County Health Department (TCHD) between January 1, 1993 and December 31, 2000. The TCHD serves the western portion of the Fort Worth-Dallas metropolitan area and includes a population of approximately 1.5 million [12]. The Fort Worth-Dallas metropolitan area is the ninth largest in the U.S. [13]. This study is part of the recent collaborative project sponsored by the Center for Disease Control and Prevention National Tuberculosis Genotyping and Surveillance Network for studying the molecular epidemiology of tuberculosis [14]. All data and materials; including isolates, isolate genotypes, demographic factors, and addresses; were collected prospectively. Moreover, one of the stated objectives of the National Tuberculosis Genotyping and Surveillance Network was to characterize places involved in potential TB transmission [15].

All positive isolates obtained from persons residing in Tarrant County were sent to the Texas Department of Health (TDH) for DNA fingerprinting. Only persons whose *M. tuberculosis *strains were typed by the Texas Department of Health Mycobacteriology Laboratory were analyzed. Clinical isolate IS6110-based RFLP analysis and spoligotyping analyses were utilized to identify patients infected with the same strain using published methods [16,17]. RFLP analysis using IS*6110 *RFLP is a powerful tool for discerning one strain of *M. tuberculosis *from another when there are greater than 6 copies of IS*6110 *however, a secondary typing method is needed to help differentiate strains with 6 or fewer IS6*110 *copies [18]. For this project, isolates were considered to be clonally related (i.e., genotypically clustered) if they had identical IS*6110 *patterns containing seven or more bands, or they had identical IS*6110 *patterns containing six or fewer bands and identical spoligotypes. A geographic cluster was defined as two or more patients with molecularly related TB strains living in Tarrant County, TX. The proportion of cases due to ongoing transmission was estimated allowing one source case per cluster (i.e. n-1 method) [5].

Any patient who did not have both spoligotyping and RFLP analysis of IS*6110 *performed on their *M. tuberculosis *isolate, and/or did not live within Tarrant County at the time of collection was excluded from the geographical analysis. Each eligible patient participated in a standard interview as part of their routine initial medical evaluation. Interview data collected included current and past employment, housing, alcohol and illicit drug use, incarceration history, sexual orientation, and psychiatric history. Persons paid daily for work were considered sporadically employed; others were employed or unemployed. Homelessness was defined as being without a permanent address for more than 3 days since 1991. If persons had a history of homelessness, paid rent by the day, or lived with a non-spousal roommate without paying rent, they were considered unstably housed. Alcoholism was defined by admission of daily consumption of three or more ounces of an alcoholic beverage; a history of alcohol-related conditions including cirrhosis, hepatitis, alcohol withdrawal seizures; or incarceration for alcohol use. Illicit drug use was defined by admission of use or documentation of being under the influence of an illicit drug. Persons were classified as having a history of incarceration if they had spent more than 24 hours in any criminal justice facility since 1991. Patients born in the U.S. or one of its territories were considered American-born; all others were considered foreign-born. All patients received HIV testing and counseling as part of standard clinical practice at the time of diagnosis. HIV status was determined from these tests.

Residential address at the time of diagnosis of tuberculosis, including zip code, were geocoded using ArcView, 4.0, Geographic Information System Software, (ESRI, Redlands, CA). After geocoding, automatically and interactively, 94% of the cases were correctly matched. The numbers of cases were then aggregated by zip code and, for each zip code, an average of the total population reported for the US Census 2000 and US Census 1990 was used to calculate incidence. Population information was retrieved from the US Census Bureau [20] and the North Central Texas Council of Governments (NCTOG) [21]. The US Census Bureau website provides census data aggregated to certain boundaries (e.g.) block groups, blocks, census tracts, zip codes, counties, and states. The NCTOG is a collection of local governments in the Dallas Fort Worth area, provides demographic and GIS data for the region. The demographic data provided has been directly extracted from the US Census. Zip-code level boundaries were established for incidence comparison purpose using zip code tabulation areas (ZTCAs) [21].

The three-dimensional analysis was performed using Inverse Distance Weighting (IDW) [22]. Interpolation is the estimation of values for points in an area not actually sampled. There are many different types of interpolation, with IDW being the simplest interpolation method. A neighborhood about the interpolated point is identified and a weighted average is taken of the observation values within this neighborhood. The weights are a decreasing function of distance. The simplest weighting function is inverse power: w(d)= 1/d^p ^with p > 0. For p = 1, the interpolated function is "cone-like" in the vicinity of the data points [22]. The resulting "cone" shows the clustering of data around the center point of a geographical area.

Statistical analysis was performed utilizing SAS V.8 statistical software (SAS Institute, Cary, NC). Patients with genotypically clustered and unique strains (non-clustered) were compared regarding each categorical risk variable by using odds ratio as a measure of association. Risk categories included patient demographics, and tuberculosis risk factors such as homelessness, HIV-infection, incarceration, and foreign birth. Because members of the clustered cases are assumed to be related, generalized estimating equations (GEE) analysis [23] was performed to determine factors associated with infection of genotypically and geographically clustered strains of *M. tuberculosis*, and to derive maximum likelihood odds ratio and 95% confidence intervals for all variables. Age was the only continuous variable. Age statistics were analyzed by comparing the means between groups. A 95% confidence interval for the mean age difference was calculated by using the normal approximation, and an independent sample two-tailed student's *t*-test was used to assess the statistical significance of the mean age difference. The institutional review board of the University of North Texas Health Science Center at Fort Worth approved this investigation.

## Results

**Figure 1:**
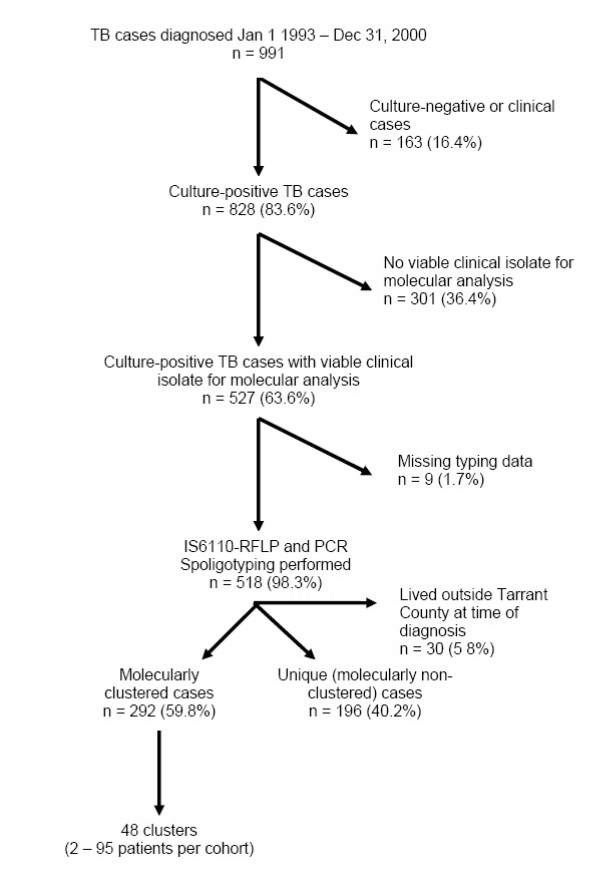
Derivation of study population, Tarrant County, Texas 1993 – 2000

From January 1, 1993 to December 31, 2000, there were 991 incident cases of tuberculosis in Tarrant County, Texas; *M. tuberculosis *was isolated from 828 (83.6%) cases. Of the 828 cases with a positive culture, 527 (63.6%) had viable clinical isolates for molecular analysis. Persons excluded because of no viable clinical isolation of *M. tuberculosis *did not differ statistically from those with those with viable isolates by age (p = 0.49), gender (p = 0.57), or location (p = 0.64). Two hundred and ninety-two (55.4%) patients met the criteria for molecular clustering. These patients were categorized into 48 clusters varying from 2 to 95 patients per group. In nine instances (1.7%), patients had an identical low copy RFLP pattern but did not have spoligotyping conducted, and were therefore classified as missing data (Figure 1). The proportion of cases attributable to ongoing transmission (allowing one source case per cluster i.e. n-1 method) is estimated to be (292 -48)/518 = 47%.

The mean age for the entire population was 44.7 ± 17.3 (SD), 44.1 ± 16.6 (SD) for those genotypically clustered, and 48.5 ± 17.6 (SD) for those with unique strains. Patients that were genotypically clustered differ significantly with age when compared to patients with unique strains, [p = 0.005]. One hundred and seventy-one (32.4%) patients were African-American, 165 (31.3%) were Caucasian, 109 (20.7%) were Hispanic, and 82 (15.6%) were Asian. African-Americans with tuberculosis were significantly more likely to have a clustered strain [OR = 2.7, 95% CI = 1.8, 4.0]. Alternatively, Asians [OR = 3.9, 95% CI = 2.3, 6.0], and Hispanics [OR = 1.9, 95% CI = 1.2, 2.9] were significantly more likely to have a unique strain. Three hundred and twenty-nine (67.4%) of the patients were males, and of these, 214 (65.0%) had clustered strains; 78 of 159 females (49.1%) had clustered strains. Males were more likely than females to have a strain that matched at least one other person in Tarrant County [OR = 1.9, 95% CI = 1.2, 2.8]. Persons with previous experience of homelessness were strongly associated with clustering suggesting a high rate of on-going transmission among this population. [OR = 12.4, 95% CI = 2.9, 52.1] (Table 1).

**Table 1:** Selected factors associated with genotypic clustering *Within Clustering, CI = confidence interval; OR = Odds Ratio

	*N*(%)*	**OR**	**95% CI**	***p*-value**
Homelessness	33 (11.3)	12.4	2.9, 52.1	*<0.001*
Living in Zip Code 1	41 (14.0)	6.2	2.4, 16.1	*<0.001*
American born	235 (80.5)	5.3	3.5, 7.9	*<0.001*
African-American	123 (42.1)	2.7	1.8, 4.0	*<0.001*
Male gender	214 (73.3)	1.9	1.3, 2.8	*0.001*
Living in Zip Code 2	40 (13.7)	1.9	1.0, 3.6	*0.038*
Living in Zip Code 3	9 (34.6)	0.3	0.1, 0.7	*0.03*

Three hundred and twenty-one (65.7%) patients were born in the United States. Of those, 235 (73.2%) had clinical isolates that matched the isolate from at least one other person living in Tarrant County. One hundred and sixty-seven patients were born outside of the United States. Of those, 57 (34.1%) clinical isolates that matched the isolate from at least one other person living in Tarrant County. U.S. born individuals were significantly more likely to be genotypically clustered than foreign-born counterparts [OR = 5.3, 95% CI 3.5, 7.9]. The birth country of foreign-born patients varied. Of those born outside of the U.S, 77 (46.1%) were born in Latin America, 47 (28.1%) in Southeast Asia, 14 (8.4%) in Sub-Saharan Africa, 12 (7.2%) in Pacific Asia, 11 (6.6%) in South Asia, and 6 (3.6%) in Europe.

**Figure 2:**
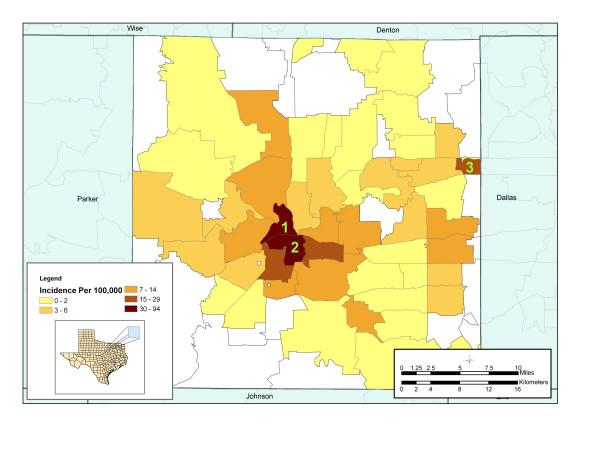
**Average incidence of Tuberculosis by zip code Tarrant County, Texas (1993 – 2000). **Specific zip codes of interest are labeled in green.

**Figure 3:**
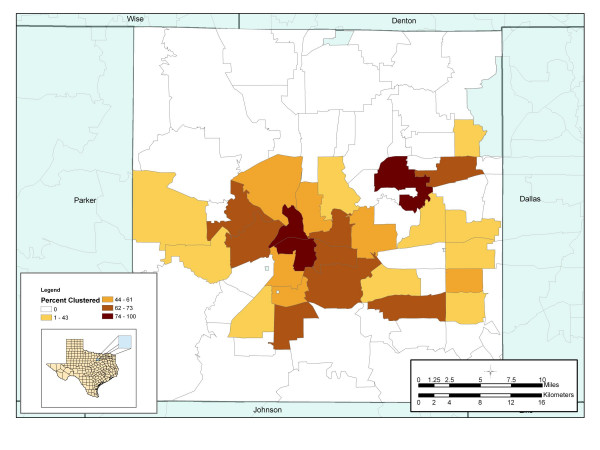
**Percent of patients genotypically cluster by zip code Tarrant County, Texas (1993 – 2000). **Percent genotypically clustered cases = number of genotypically clustered cases/ total number of cases × 100

Evaluation of the spatial distribution of number of cases within zip-code boundaries displayed a distinct geographical distribution of disease. The average incidence for the entire county during the study period was 5.9 cases per 100,000. Zip code 1 recorded the highest incidence of 94.3 cases per 100,000 populations, followed by zip code 2 with an average incidence of 55.2 cases per 100,000 population (Figure 2). These areas are characterized by low socioeconomic status, high unemployment rates, homelessness, drug use, and poor quality housing conditions. To examine how molecular clustering varies spatially by zip code, a map of percent molecular clustering at the zip-code level was produced (Figure 3). This map displayed the number of genotypically clustered cases divided by the total number of cases reported in that zip code. The map demonstrated that molecularly clustered disease is not homogenously distributed throughout the county.

GIS analysis demonstrated that the areas with the highest incidence also have the highest proportion of persons with genotypically clustered isolates. A strong preponderance of clustering occurred in the urban center of Tarrant County. The highest proportion of persons with molecular clustered TB isolates (80.4% clustered) occurred in the same zip code with the highest incidence. Similarly, zip code 2 on the southeast border of zip code 1, recorded the second highest proportion of persons with molecular clustered TB isolates with 76.6% of all reported cases clustered. Cases reported in zip code 1 were more than six times as likely [OR = 6.2, 95% CI = 2.4, 16.1] than any other zip code to have isolates that match at least one other person living in Tarrant County.

In zip code 3, we observed a morbidity that was more than triple the county average (22.3 cases per 100,000). Unlike other high morbidity areas, zip code 3 had a strong preponderance of unique strain distribution. In this zip code, 17 out of 26 (65.4%) patients had isolates that did not match any other patient in Tarrant County. Cases reported in this zip code were 70% less likely [OR = 0.3, 95% CI = 0.1, 0.7] to have a clustered strain, suggesting that the high rates of tuberculosis did not result from local on-going transmission.

## Discussion

The number of cases in the United States is at its lowest point in history, with 15,075 cases reported in 2002 [24]. The role of treatment of LTBI in tuberculosis elimination is of increasing importance. The IOM recommended developing improved methods for identifying persons with recently acquired infections as an important component of strategic tuberculosis elimination in the United States [4]. This study uncovered geographical links to on-going tuberculosis transmission enhancing traditional public health surveillance. We found that by combining molecular strain characterization with GIS analysis that risk of on-going transmission was geographically focal (p = 0.003) with significant clustering of cases occurring in 3 of 59 zip codes. This demonstrated that the current methods of surveillance of contacts of persons with tuberculosis were not completely effective in interrupting disease transmission in these zip code areas.

**Figure 4:**
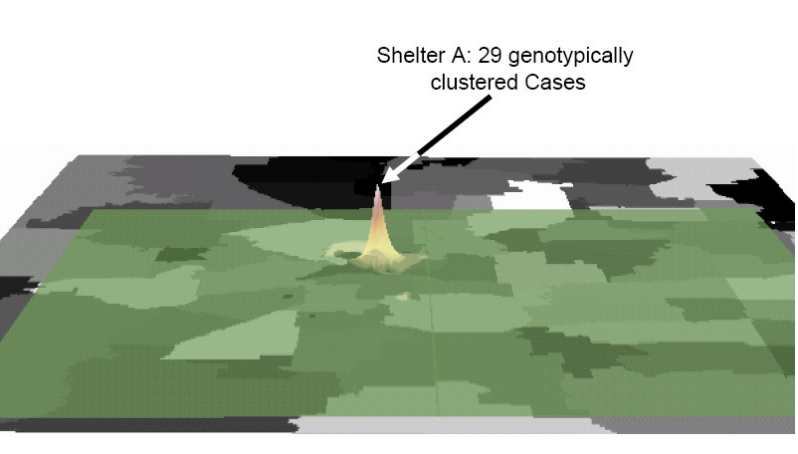
**Three-dimensional analysis using Inverse Distance Weighting Interpolation, Tarrant County, Texas (1993 – 2000). **Inverse power: w(d)= 1/d^p ^with p = 1. Zip code 1 consists of 264.89 acres.

The use of molecular strain characterization methods in conjunction with traditional surveillance has led to the recognition of a number of risk factors associated with on-going transmission, and has identified numerous outbreaks of tuberculosis undetected by conventional approaches [25-27]. These studies have demonstrated the importance of non-household location based transmission, such as homeless shelters, and social settings such as bars and crack houses [26-29]. Urban centers have traditionally had higher rates of tuberculosis than rural areas [30,31]. Population density, poverty and overcrowding appear in most areas to be major factors for disease transmission [32]. We found similar risks in our population. Location factors, specifically where patients reside at the time of diagnosis, were found to be significantly associated for certain zip codes in Tarrant County. Cases in urban zip codes 1 [OR = 6.2; 95% CI = 2.4, 16.1] and 2 [OR = 1.9; 95% CI = 1.3, 2.8] were strongly associated to infection with a clustered strain as compared to the rest of the county. These zip codes are in the urban center of the county. Zip code 1 is also the site of the largest homeless shelter in the county. Inverse Distance Weighting of this area graphically (Figure 4) demonstrates the three-dimensional result of the interpolation, representing the burden of disease in this area. The resulting "cone" shows the geographic clustering of cases around a particular point of the zip code, the physical location of homeless shelter.

These finding are similar to those reported in Los Angeles where locations, specifically homeless shelters were identified as important sites of tuberculosis transmission [33]. Similarly, in Houston, locations, specifically bars, were as important as persons in uncovering epidemiological and genotypical links in outbreak investigations [34]. The authors of both of these studies suggested measures to reduce tuberculosis transmission should be based on locations as well as personal contacts [33,34].

We identified that 55% of our patients were clustered and 47% attributable to ongoing community transmission. This differs from a study conducted in a high incidence area of South Africa, where 72% of cases were clustered and 58% attributable to ongoing community transmission [11]. Our lower percentage of clustering and attributable on-going transmission may be related to a much lower reported overall morbidity or the effects of differences in programmatic interventions, such as contact investigation, targeted screening efforts or DOT completion rates.

Although the majority of the tuberculosis morbidity within the developed world is strongly influenced by imported tuberculosis from high prevalence countries [35,36], the rates at which these individuals transmit disease to the general population remain low. We found that foreign-born cases were significantly more likely to have a unique strain [OR = 6.4, 95% CI = 4.1, 9.8] indicating that immigrants were less likely to be source of ongoing transmission of TB in Tarrant County. In a San Francisco based study, investigators identified only two instances of a foreign-born individual transmitting the disease to the native population [37]. Similarly, only 1.8% of transmission from infectious Somali immigrants was to the native population in the Netherlands over the period from 1992 to 1999 [38]. Historically, tracking these populations of foreign born to assess transmission has been difficult. GIS provides another approach for evaluating this issue. As this study illustrates, identifying geographical areas of increased incidence with a high percentage of unique strains may improve local surveillance methods to locate hard to reach foreign-born populations before transmission occurs.

There are some limitations to this research approach. This is based on secondary data, which includes variables collected from a cross-sectional period of time. Although each case is an incident case at the time of diagnosis, under this cross-sectional design, exposure and disease outcomes are assessed simultaneously. In addition patients with tuberculosis may have moved shortly before their diagnosis. However, this should not cause systematic error (bias) or result in an association of clustering with specific locations, because these events would be expected to produce a random misclassification. Also persons exposed within certain zip codes may go on to reside elsewhere and later develop the disease, and result in an underestimate of the morbidity and that may be reflected in calculating associations. Finally, genotyping results were not available for a proportion of TB cases in this study. Some unique isolates might have clustered if some of the missing isolates had been available or if other cases with the same strain moved or are located outside the study area [39]. We therefore believe that estimates of the degree of clustering and the size of clusters are conservative.

When using this approach TB control programs must select the appropriate geographical boundary to examine transmission in their area. For example, using zip codes may be too large a boundary in very populated metropolitan areas. Census block groups may provide greater resolution in determining localized transmission.

Nor are the molecular techniques used without limitation. Patients are clustered according to their isolates having the same genotype. While IS*6110 *RFLP is recognized as the most discriminatory method for genotyping *M. tuberculosis *isolates, the discriminatory ability of the technique decreases when there are fewer than 6 IS*6110 *insertions in the genome. In this case, spoligotyping was used for further strain discrimination. However, it is still possible that some isolates classified as being the same strain based on identical genotypes may represent distantly related, but distinct, strains. Moreover, demonstration that particular patients have the same strain supports, but does not irrefutably prove, direct transmission between these patients as opposed to another source of infection. Conversely, strains continue to evolve, and the resulting genotypic differences over time can result in assigning isolates from cases of direct transmission to distinct strain lineages. Given that a small minority of the isolates had fewer than 6 IS*6110 *bands (18.2%) or differed by the presence or absence of one band in an otherwise conserved pattern (3.7%), we believe that estimates of the degree of clustering and the size of clusters are conservative.

## Conclusion

Using GIS analysis combined with molecular epidemiological surveillance can be an effective method for identifying tuberculosis transmission not identified during standard contact tracing methods. The application of these methods can be utilized in countries where contact tracing is routinely performed. These methods can enhance targeted screening and control efforts, with the goal of interruption of disease transmission and ultimately incidence reduction. This study demonstrates that using existing health data, GIS can identify previously undetected TB transmission. These results were used to design new targeted screening efforts [40]. Studies of these efforts are ongoing to demonstrate if identifying focal areas for targeted screening has utility in reducing TB transmission.

## List of abbreviations

CI Confidence Interval

GEE Generalized Estimating Equations

GIS Geographic Information Systems

HIV Human Immuno-deficiency Virus

IDW Inverse Distance Weighting

NCTCG North Central Texas Council of Governments

OR Odds Ratio

RFLP Restriction Fragment Length Polymorphism

TB Tuberculosis

TCHD Tarrant County Health Department

TDH Texas Department of Health

## Authors' Contributions

Study concept and design: PM, MB, SW

Acquisition of data: PM, TQ, KJ, DD, GB, SW

Analysis and interpretation of data: PM, MB, TQ, JO, SW

Drafting of the manuscript: PM, SW

Critical revision of the manuscript for important intellectual content: SW, PM, JO, TN

Statistical expertise: KS, MB, PM

Obtained funding: TQ, SW

Administrative, technical or material support: GB

## Funding/Support

This work was supported in part by the Centers for Disease Control and Prevention, National Tuberculosis Genotyping and Surveillance Network Cooperative Agreement U52/CCU600497-18, and Tuberculosis Epidemiologic Studies Consortium 200-2001-00084.
